# DNA methyltransferase 3A isoform b contributes to repressing *E-cadherin* through cooperation of DNA methylation and H3K27/H3K9 methylation in EMT-related metastasis of gastric cancer

**DOI:** 10.1038/s41388-018-0285-1

**Published:** 2018-05-02

**Authors:** He Cui, Ying Hu, Didi Guo, Aifeng Zhang, Yuejun Gu, Shaodan Zhang, Chengcheng Zhao, Pihai Gong, Xiaohui Shen, Yiping Li, Huazhang Wu, Ling Wang, Zhujiang Zhao, Hong Fan

**Affiliations:** 1 Department of Medical Genetics and Developmental Biology, Medical School, The Key Laboratory of Developmental Genes and Human Diseases, Ministry of Education, Southeast University, 210009 Nanjing, China; 2 Jiangsu Province Hospital and Nanjing Medical University First Affiliated Hospital, 210029 Nanjing, China; 3 Department of Pathology, Medical School, Southeast University, 210009 Nanjing, China

## Abstract

DNA methyltransferase 3A (DNMT3A) has been recognised as a key element of epigenetic regulation in normal development, and the aberrant regulation of DNMT3A is implicated in multiple types of cancers, especially haematological malignancies. However, its clinical significance and detailed functional role in solid tumours remain unknown, although abnormal expression has gained widespread attention in these cancers. Here, we show that DNMT3A isoform b (DNMT3Ab), a member of the DNMT3A isoform family, is critical for directing epithelial–mesenchymal transition (EMT)-associated metastasis in gastric cancer (GC). DNMT3Ab is positively linked to tumour-node-metastasis (TNM) stage, lymph node metastasis and poor prognosis in GC patients. Overexpression of DNMT3Ab promotes GC cell migration and invasion as well as EMT through repression of *E-cadherin*. Meanwhile, DNMT3Ab promotes lung metastasis of GC in vivo. Mechanistic studies indicate that DNMT3Ab mediates the epigenetic inaction of the *E-cadherin* gene via DNA hypermethylation and histone modifications of H3K9me2 and H3K27me3. Depletion of DNMT3Ab effectively restores the expression of *E-cadherin* and reverses TGF-β-induced EMT by reducing DNA methylation, H3K9me2 and H3K27me3 levels at the *E-cadherin* promoter. Importantly, DNMT3Ab cooperated with H3K9me2 and H3K27me3 contributes to the transcriptional regulation of *E-cadherin* in a Snail-dependent manner. Further, gene expression profiling analysis indicates that multiple metastasis-associated genes and oncogenic signalling pathways are regulated in response to DNMT3Ab overexpression. These results identify DNMT3Ab as a crucial regulator of metastasis-related genes in GC. Targeting the DNMT3Ab/Snail/*E-cadherin* axis may provide a promising therapeutic strategy in the treatment of metastatic GC with high DNMT3Ab expression.

## Introduction

Gastric cancer (GC) is one of the most common fatal malignancies worldwide [1]. The prognosis of GC is poor, and the 5-year survival rate of patients with stage IV disease remains nearly 25% [2, 3]. Similar to other cancers, metastasis in GC is a high potential risk in clinical practice and accounts for a major source of recurrence and mortality [4, 5]. Genetically, the accumulation of epigenetic alterations is a frequent event during the process of metastasis [6]. Multiple modes of deregulation, including aberrant DNA methylation and histone modification, are known to be implicated in metastatic GC [7, 8]. Thus, a better understanding of the precise epigenetic mechanisms underlying metastasis is critical to provide novel therapeutic strategies for GC patients.

DNA methylation provides a stable repression mark that modulates the expression of tumour suppressor genes (TSGs) and is mediated by a family of enzymes called DNA methyltransferases (DNMTs), which includes three known active DNMTs in mammals: DNMT1, DNMT3A and DNMT3B [9, 10]. Previous data have shown that DNMTs are upregulated in various tumours [10, 11]. Recently, significant progress has been made in the understanding of DNMT3A in GC. Most importantly, increased DNMT3A expression is closely linked to the poor survival rate of GC patients but not for patients with other cancers, including breast cancer, lung cancer and liver cancer (analysis from the Kaplan–Meier plotter website). Moreover, several pieces of evidence suggest that the de-regulation of DNMT3A may be more important for GC progression than that of DNMT1 and DNMT3B. Yang et al. found that the magnitude of DNMT3A overexpression in GC is higher than that of DNMT1 and DNMT3B, and high DNMT3A expression alone is closely correlated with tumour-node-metastasis (TNM) stage and lymph node metastasis of GC cells [12]. Cao et al. found that the poor survival rate of GC patients is associated with elevated DNMT3A but not DNMT1 or DNMT3B expression [13]. Additionally, our previous study reported that a functional polymorphism in the DNMT3A promoter contributes to the incidence of GC [14]. Collectively, these data suggest that DNMT3A may be a critical contributor in GC.

DNMT3A is well known to be implicated in normal development, and genetic mutations in DNMT3A are closely associated with acute myeloid leukaemia (AML) [15–17]. However, the potential roles of DNMT3A in solid tumours, especially in metastatic tumours, are largely unknown. Previous studies have found that the methyI-H3K9-binding protein MPP8 enhances tumour cell motility and invasion properties by interacting with DNMT3A [18]. High-mobility group A2 protein, HMGA2, fosters the binding of DNMT3A to the *E-cadherin* promoter and the removal of epithelial features by tumour cells [19]. Similarly, DNMT3A is involved in the feedback loop between miR-124 and the TGF-β pathway in non-small cell lung cancer metastasis [20]. Very recently, Pistore et al. indicated that DNMT3A-dependent DNA methylation is essential for the epithelial–mesenchymal transition (EMT) in prostate cancer cells [21], which supports our finding that dysregulation of DNMT3A and miR-29b/c led to *E-cadherin* repression and promoted GC cell migration and invasion [22]. Notably, DNMT3A consists of at least two functional proteins with catalytic activity, including a full-length isoform a (DNMT3Aa, also known as DNMT3A1 in some reports) and an identified short isoform b (DNMT3Ab, also known as DNMT3A2 in some reports) [15]. DNMT3Ab is initiated from a different promoter in the sixth intron of the *DNMT3A* gene, which encodes DNMT3Aa. Both DNMT3Aa and DNMT3Ab contain two defined domains: the PWWP domain and the ADD domain, but an additional 223 amino acid N-terminal domain is present only in DNMT3Aa [23]. Suetake et al. indicated that this N-terminus is important for the DNA-binding activity of DNMT3A [24]. In addition, DNMT3Ab is the predominant isoform expressed in embryonic stem cells and embryonal carcinoma cells, and its expression is correlated with high de novo methylation activity [23]. These data motivated us to re-assess DNMT3A and further investigate which of the individual isoforms or combinations of isoforms are the key factors in relevant malignant phenotypes.

In this study, we analysed patient samples along with in vitro and in vivo functional studies to identify a critical role of DNMT3Ab in GC prognosis and metastasis. The underlying mechanism is mediated by the repression of *E-cadherin* through the recruitment of DNMT3Ab to its promoter. DNMT3Ab cooperated with H3K9me2 and H3K27me3 to regulate *E-cadherin* expression and influence the metastatic ability of GC cells in a Snail-dependent manner. These results represent a significant step forward in understanding the contribution of DNMT3A and its isoforms to GC metastasis and in providing a potential target for epigenetic-based GC therapy.

## Results

### Overexpression of DNMT3Ab and its clinical significance in GC

**Fig. 1:**
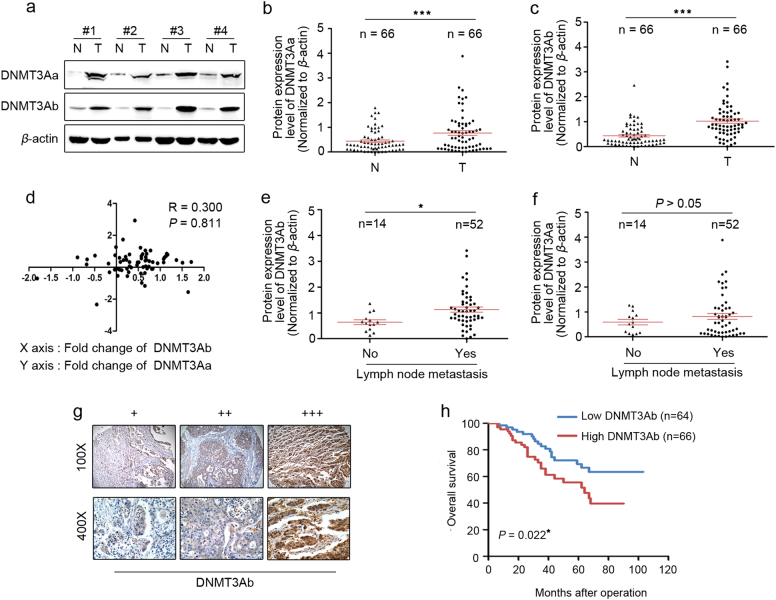
The expression of DNMT3Ab detected in GC tissues. **a** Western blot analysis of DNMT3Aa and DNMT3Ab in four representative paired adjacent non-tumour (N) tissues and GC (T) tissues. β-actin was used as a loading control. The band intensities of each sample were quantified with *ImageJ* software and normalised to β-actin intensities, and the values represent relative protein levels. **b**, **c** Relative protein levels of DNMT3Aa or DNMT3Ab in 66 paired adjacent non-tumour (N) tissues and GC (T) tissues. In both panels, the red lines are the mean ± SEM (***P < 0.001). **d** The correlation between DNMT3Ab and DNMT3Aa protein levels in 66 GC clinical samples was not significant (*R* = 0.300, *P* > 0.05). **e**, **f** Relative protein levels of DNMT3Ab and DNMT3Aa in 66 GC clinical samples with or without metastasis (*P < 0.05). **g** The expression of DNMT3Ab was detected by IHC staining in 130 GC tissues. (+) denotes low expression, (++, +++) denotes high expression. **h** Kaplan–Meier analysis of the correlation between DNMT3Ab expression and overall survival in 130 patients with GC (*P < 0.05)

Previously, we demonstrated the contribution of DNMT3Aa in the promotion of GC proliferation [25]. To test the different roles of the truncated DNMT3Ab versus DNMT3Aa, we first analysed DNMT3Aa and DNMT3Ab expression by Western blot in a cohort of 66 paired GC specimens (Fig. 1a). The results showed that DNMT3Aa and DNMT3Ab were dramatically overexpressed in tumour tissues compared with those in paired adjacent non-tumour tissues (Fig. 1b, c), and upregulation of DNMT3Aa and DNMT3Ab (defined as a >2-fold increase) was detected in 37/66 (56.1%) and 41/66 (62.1%) GC tissue samples (Supplementary Figure S1a and b). However, correlation analysis showed that DNMT3Ab expression was not related to DNMT3Aa expression (Fig. 1d). A clinicopathologic association analysis in 66 GCs revealed that high DNMT3Ab expression was significantly related to increased TNM stage and more positive lymph nodes, whereas high DNMT3Aa expression was strongly associated with tumour cell differentiation and vascular invasion (Table 1 and Supplementary Table S1). Moreover, analysis of the primary tumour tissue showed that patients with lymph node metastasis had higher tumour DNMT3Ab levels than did those without metastasis, whereas no significant correlation was found between the expression of DNMT3Aa and lymph node metastasis (Fig. 1e, f). These findings suggested that DNMT3Ab may contribute to metastasis in patients with GC. Next, we analysed DNMT3Aa and DNMT3Ab protein levels by immunohistochemistry (IHC) staining in sections from 130 paraffin-embedded GC specimens. DNMT3Aa was located mainly in the nucleus of GC cells (Supplementary Figure S1c), and DNMT3Ab was located diffusely throughout the nucleus and cytoplasm of GC cells (Fig. 1g). Strong DNMT3Ab staining (++; +++) was positively correlated with TNM stage and lymph node metastasis status (Supplementary Table S2). Higher expression of DNMT3Ab, but not DNMT3Aa, was significantly correlated with poorer overall rates (Fig. 1h and Supplementary Figure S1d). Multivariate Cox hazard analysis revealed that DNMT3Ab is an independent and significant risk factor for reduced survival (Table 2). Together, these results indicate that DNMT3Ab may be an important factor involved in GC prognosis and metastasis.

**Table 1:** Clinicopathological correlation of DNMT3Ab expression in GC tissues

Feature	T > N	T ≤ N	*P*-value
Age	60.48 ± 9.79	58.10 ± 12.07	0.408
Gender (*n* = 66)			
Female	13	4	
Male	28	21	0.157
Diameter(*n* = 64)			
≤5 cm	21	15	
>5 cm	19	9	0.435
Lauren (*n* = 56)			
Diffuse type	20	10	
Intestinal type	17	9	0.920
Histologic grade (*n* = 63)			
Poor	22	13	
Moderate	18	10	
High^a^	0	0	0.907
TNM staging (*n* = 65)			
Stage I/II	11	13	
Stage III/IV	30	11	0.028*
Vascular invasion (*n* = 60)			
Yes	9	1	
No	30	20	0.069
Lymph node metastasis (*n* = 66)			
Yes	37	15	
No	4	10	0.004*

*N* non-tumour tissues, *T* tumour tissues

*Significant differences are shown in bold

^**a**^No cases in this pathological classification

**Table 2:** Multivariate Cox regression analysis of potential prognostic factors for patients with GC

Variable	RR (95% CI)	*P*-value
Age	1.030 (0.999–1.063)	0.060
Gender		
Female	1.000	
Male	0.798 (0.405–1.570)	0.513
Diameter		
≤5 cm	1.000	
>5 cm	2.290 (1.243–4.216)	0.008*
Lymph node metastasis		
No	1.000	
Yes	6.120 (1.888–19.836)	0.003*
DNMT3Ab expression		
Low	1.000	
High	2.577 (1.409–4.713)	0.002*

*Significant differences are shown in bold

### DNMT3Ab promotes GC cell metastasis

To explore the oncogenic function of DNMT3Ab in GC cells, we first found that endogenous DNMT3Ab expression was upregulated in a panel of GC cell lines compared with one immortalised gastric cell line GES-1 (Supplementary Figure S2a). Next, we analysed the metastatic potential of GC cell lines. The results revealed that MKN45 and BGC-823 cell lines had weak metastatic potential, while MKN28 and MCG-803 cell lines displayed strong metastatic potential (Supplementary Figure S2b), which is supported by previous reports [26, 27]. Thus, we selected MKN45 and BGC-823 to establish DNMT3Ab stable overexpression cells and MKN28 to establish stable knockdown cells using two independent shRNAs against DNMT3Ab. DNMT3Ab expression in the above cells was confirmed by Western blot (Supplementary Figure S2c and d).

**Fig. 2:**
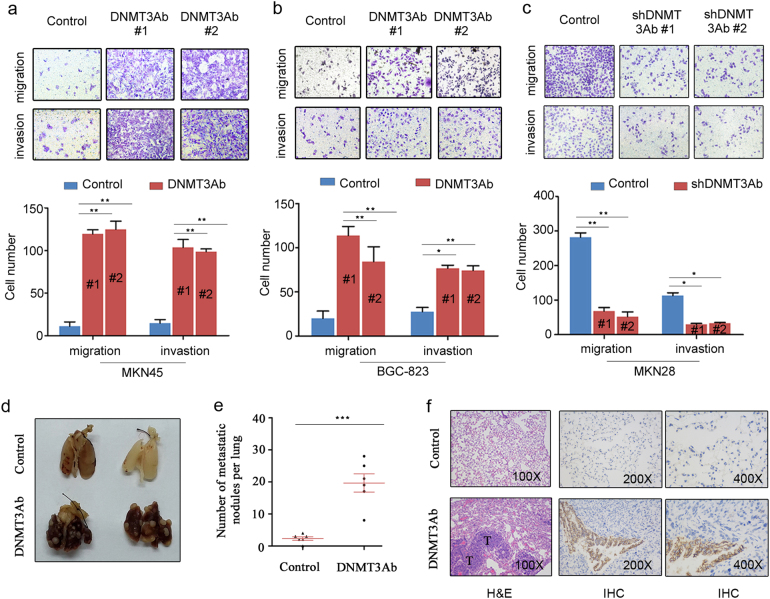
DNMT3Ab increases cell motility in vitro and metastasis in vivo. **a**–**c** Representative images (top) and relative bar graphs (bottom) depicting the migration and invasion of DNMT3Ab-transfected MKN45 and BGC-823 cells and the DNMT3Ab-knockdown MKN28 cells. The number of cells that migrated or invaded was counted in five fields. The migration and invasion rates are presented as the number of cells per field (*P < 0.05; **P < 0.01). **d** Metastatic nodules on the surface of the lung. **e** The number of nodules on the lungs of mice was quantified (*n* = 6 per group) 6 weeks after injection of DNMT3Ab-tranfected or control MKN45 cells into the tail vein. Data are shown as the mean ± SEM (***P < 0.001). **f** Serial sections of metastatic tumours and normal lung were stained with H&E. DNMT3Ab protein in the metastatic tumours and normal lung was detected by IHC

Clinical association analysis revealed that higher expression of DNMT3Ab was significantly associated with GC metastasis. To investigate the effects of DNMT3Ab on the metastatic ability of GC cells, in vitro and in vivo assays were performed. As shown in Fig. 2a, b, DNMT3Ab overexpression significantly increased MKN45 and BGC-823 cell migration and invasion. Wound healing assays showed that MKN45 and BGC-823 cells with increased DNMT3Ab expression displayed a faster recovery time than did control cells (Supplementary Figure S3a). Specifically, DNMT3Ab-tranfected cells did not exhibit notable differences in cell proliferation, as assessed by foci formation analysis (Supplementary Figure S3b). Conversely, silencing DNMT3Ab in MKN28 cells dramatically decreased the migratory and invasive abilities of tumour cells (Fig. 2c and Supplementary Figure S3c). Similarly, we inhibited the expression of DNMT3Ab by siRNAs transfection in MCG-803 cell lines and found that silencing DNMT3Ab reduced the migratory and invasive abilities of these cells (Supplementary Figure S3d). However, the effect of DNMT3Ab knockdown on the migration and invasion of MKN45 and BGC-823 cells showed no significant changes, which may be due to low metastatic potential of these cells (Supplementary Figure S3e and f). Furthermore, an in vivo metastasis assay showed that DNMT3Ab overexpression increased the incidence of lung metastasis and the number of metastatic nodules compared with those of the control groups (Fig. 2d, e). The metastatic lesions in lungs were confirmed by histological analysis, and higher DNMT3Ab protein expression was observed in the xenografts by IHC (Fig. 2f). Collectively, these results indicate that DNMT3Ab promotes GC migration and invasion in vitro and metastasis in vivo.

### DNMT3Ab expression induces EMT

**Fig. 3:**
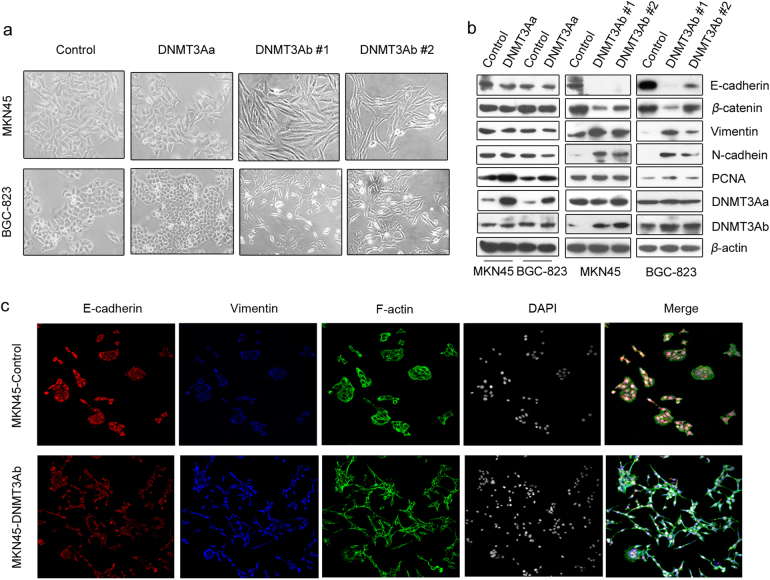
DNMT3Ab induces the process of EMT. **a** Representative images of tumour cell morphology in DNMT3Aa- and DNMT3Ab-tranfected MKN45 and BGC-823 cells. **b** Western blot was used to show the expression of epithelial markers (E-cadherin and β-catenin) and mesenchymal markers (Vimentin and N-cadherin) in DNMT3Aa- or DNMT3Ab-transfected cells relative to that in the control cells. PCNA was used as a positive control. **c** Redistribution of actin filaments to filopodium-like structures in DNMT3Ab- transfected MKN45 cells, which were stained for F-actin. Double IF staining of E-cadherin and Vimentin was performed in DNMT3Ab-tranfected MKN45 cells. The cells were counterstained with DAPI to visualise the nuclei

While investigating the potential roles of DNMT3Ab in vitro, we unexpectedly observed that the morphology of DNMT3Ab-transfected cells changed from epithelial-like to fibroblast-like in phase-contrast images, suggesting that these cells underwent EMT. In contrast, neither of the DNMT3Aa-transfected cells showed obvious changes in GC cell morphology (Fig. 3a). Moreover, we found that the epithelial markers E-cadherin and β-catenin were decreased, while the mesenchymal markers Vimentin and N-cadherin were increased in DNMT3Ab-transfected GC cells compared with those in the control cells (Fig. 3b). Conversely, silencing DNMT3Ab expression prevented EMT (Supplementary Figure S3g and h). Interestingly, increased DNMT3Aa had no significant effects on EMT marker expression (Fig. 3b). To confirm the effect of DNMT3Ab on EMT, immunofluorescence staining was performed to detect the expression of E-cadherin and Vimentin. As shown in Fig. 3c, decreased E-cadherin and increased Vimentin expression patterns were found in DNMT3Ab-transfected MKN45 cells. F-actin staining revealed increased filopodium formation, suggesting that rearrangement of the actin cytoskeleton occurred in these cells. Taken together, these data indicate that DNMT3Ab enhances the invasiveness of GC cells, probably by inducing EMT.

### *E-cadherin* is repressed by DNMT3Ab-mediated DNA methylation

**Fig. 4:**
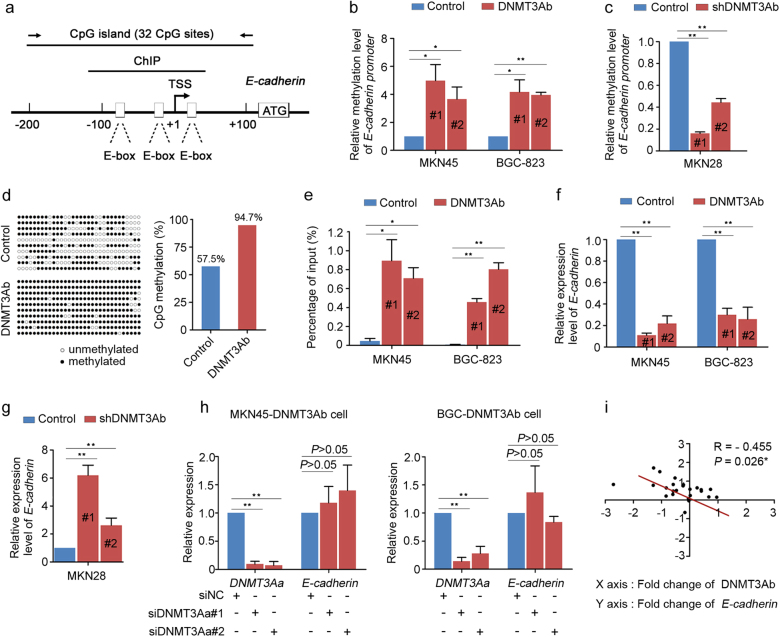
DNMT3Ab represses *E-cadherin* expression. **a** Diagram of the *E-cadherin* gene promoter with the transcription start site (TSS) indicated. One region (−187 bp to +177 bp) spanning a CpG island with 32 CpG sites was analysed. The short black line represents the location of the fragment detected by the ChIP assay. The E-box elements near the TSS are indicated. **b**, **c** Methylation levels at the *E-cadherin* promoter were detected by Q-MSP assays in DNMT3Ab-transfected MKN45 and BGC-823 cells, and DNMT3Ab knockdown MKN28 cells (*P < 0.05). **d** Methylation status of the *E-cadherin* promoter in DNMT3Ab-tranfected MKN45 cells relative to that in control cells, detected by BGS assay (left). Thirty-two individual CpG sites within the CpG island (from 187 to +177 bp) were sequenced. Each row represents a single sequence; ○ indicates unmethylated CpG sites and ● indicates methylated CpG sites. The bar graphs depict the *E-cadherin* promoter methylation rates (right). **e** The binding of DNMT3Ab to the *E-cadherin* promoter was detected by ChIP analysis in DNMT3Ab-tranfected cells (*P < 0.05, **P < 0.01). **f**, **g** The relative expression of *E-cadherin* was detected in DNMT3Ab-tranfected or -knockdown cells using qPCR. β-actin was used as an internal control (**P < 0.01). **h** qPCR was performed to determine the *E-cadherin* mRNA levels in DNMT3Ab-tranfected cells after transient transfection of siRNA targeting DNMT3Aa. β-actin was used as an internal control (**P < 0.01). **i** The correlation between DNMT3Ab and *E-cadherin* expression in 24 clinical samples (*R* = −0.455, *P < 0.05)

It is widely acknowledged that the hallmark of EMT is the functional loss of *E-cadherin* [28]. Previous data have indicated that repression of *E-cadherin* by DNA methylation is closely correlated with metastasis in GC [29]. We first determined the baseline levels of DNA methylation on *E-cadherin* promoter among GC cell lines (Supplementary Figure S4a) and then evaluated whether DNMT3Ab-mediated DNA methylation is required for *E-cadherin* transcription (Fig. 4a). The results showed that DNMT3Ab overexpression increased methylation levels at the *E-cadherin* promoter, whereas marked de-methylation of *E-cadherin* promoter was found in DNMT3Ab-knockdown cells (Fig. 4b, c; Supplementary Figure S4b and c). Next, bisulphite DNA sequencing (BGS) analysis was performed and demonstrated that the frequency of *E-cadherin* promoter methylation was 94.7% in DNMT3Ab-transfected cells, which was much higher than the 57.5% frequency measured in control cells (Fig. 4d), suggesting that DNMT3Ab-mediated DNA methylation is involved in the regulation of *E-cadherin* expression. Chromatin immunoprecipitation (ChIP) analysis revealed enhanced binding of DNMT3Ab (Fig. 4e), but not DNMT3Aa, to the *E-cadherin* promoter after DNMT3Ab overexpression (Supplementary Figure S4d). Meanwhile, the expression of *E-cadherin* was analysed in cells with overexpression or knockdown of DNMT3Ab. The results found that increased DNMT3Ab led to reduced *E-cadherin* expression, while decreased DNMT3Ab increased *E-cadherin* expression (Fig. 4f, g; Supplementary Figure S4e). Notably, no obvious de-repression of *E-cadherin* was observed after silencing DNMT3Aa in DNMT3Ab-transfected cells (Fig. 4h and Supplementary Figure S4f). Furthermore, we analysed the expression of *E-cadherin* in 24 paired GC samples (Supplementary Figure S4g), and we found a negative correlation between DNMT3Ab and *E-cadherin* expression but no correlation between DNMT3Aa and *E-cadherin* expression (Fig. 4i and Supplementary Figure S4h). Altogether, these results suggest that DNMT3Ab-mediated DNA methylation contributes to the transcriptional silencing of *E-cadherin*.

### Inhibition of DNMT3Ab reduces EMT and metastasis of GC cells

**Fig. 5:**
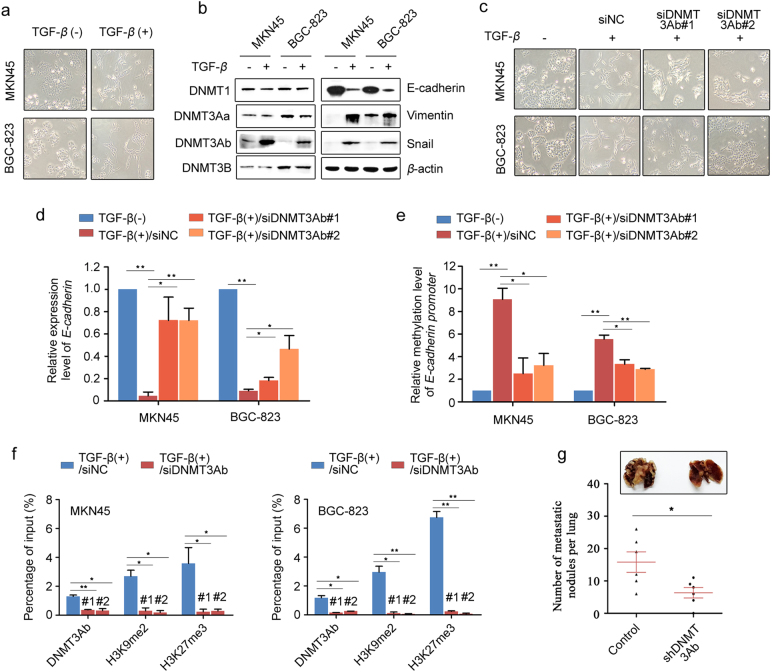
Silencing DNMT3Ab expression reverses the EMT process and inhibits metastasis. **a**, **b** MKN45 and BGC-823 were treated with TGF-β (2 ng/ml) for 14 days. Cell morphology changes associated with EMT phenotype. Expression of DNMT1, DNMT3Aa, DNMT3Ab, DNMT3B, E-cadherin, Vimentin and Snail in these cells was detected by Western blot. β-actin was used as a loading control. **c** DNMT3Ab or negative control siRNAs were transiently transfected into MKN45 and BGC-823 cells, followed by mock or TGF-β (2 ng/ml) treatment for 3 days. Cell morphology changes associated with the EMT phenotype. **d** MKN45 and BGC-823 cells were treated as described in **c**. The expression of *E-cadherin* was detected using qPCR. β-actin was used as an internal control. The values indicate the mean ± SD of three independent experiments. **e** MKN45 and BGC-823 cells were treated as descripted in **c**. The methylation levels at the *E-cadherin* promoter were detected by Q-MSP. The values indicate the mean ± SD of three independent experiments. **f** MKN45 and BGC-823 cells were treated as descripted in **c**. DNTM3Ab, H3K9me2 and H3K27me3 at the *E-cadherin* promoter were analysed by ChIP-qPCR. **g** The presence of metastatic nodules on the surface of the lung. The number of nodules on the lungs of mice was quantified (*n* = 6 per group) 6 weeks after the injection of DNMT3Ab-knockdown MKN28 cells or control cells into the tail vein. The data are shown as the mean ± SEM (*P < 0.05)

Having established the roles of DNMT3Ab in *E-cadherin* repression, we then examined whether DNMT3Ab is required for the process of EMT. As expected, GC cell lines treated with TGF-*β* acquired mesenchymal morphology, downregulated the epithelial marker E-cadherin, upregulated the mesenchymal marker Vimentin and showed the induction of the EMT-related factor Snail (Fig. 5a, b). Intriguingly, we detected significant changes in endogenous DNMT3Ab, but not DNMT1, DNMT3Aa or DNMT3B, after exposure to TGF-β for 14 days or 3 days in both GC cell lines tested (Fig. 5b and Supplementary Figure S5a). Next, ChIP analysis for DNMT3Aa and DNMT3Ab from the TGF-β*-*treated cells was used to directly show that DNMT3Ab, but not DNMT3Aa, is recruited to the *E-cadherin* promoter (Supplementary Figure S5b). Moreover, we found that silencing DNMT3Ab blocked TGF-β-induced EMT in MKN45 and BGC-823 cells (Fig. 5c). The above results suggested that DNMT3Ab may play an important role in EMT. To confirm this causal relationship, we found that silencing DNMT3Ab increased *E-cadherin* and was accompanied by decreased DNA methylation of the *E-cadherin* promoter in TGF-β treatment cells (Fig. 5d, e). Because DNA methylation is frequently linked to the acquisition of repressive histone methylations, we performed ChIP analysis to evaluate whether H3K9me2 and H3K27me3 were involved in the DNMT3Ab-mediated regulation of *E-cadherin*. Baseline levels of H3K9me2 and H3K27me3 status at the *E-cadherin* promoter were tested in the GC cells used in this study (Supplementary Figure S5c), and we found that less binding of DNMT3Ab coincided with a remarkable reduction of H3K9me2 or H3K27me3 status at the *E-cadherin* promoter in TGF-β-induced GC cells (Fig. 5f), suggesting that DNMT3Ab facilitated histone methylation at the *E-cadherin* promoter. Furthermore, metastases experiments indicated a reduced metastatic frequency in nude mice injected with DNMT3Ab knockdown cells (Fig. 5g). Together, these results indicate that DNMT3Ab-mediated regulation of *E-cadherin* expression is required for EMT in GC cells.

### DNMT3Ab regulates *E-cadherin* in a Snail-dependent manner

**Fig. 6:**
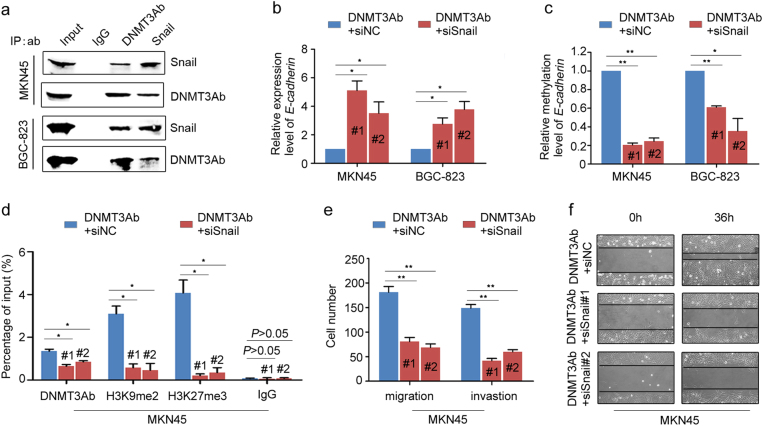
Snail is required for DNMT3Ab-mediated *E-cadherin* silencing. **a** Endogenous DNMT3Ab and Snail were immunoprecipitated from MKN45 and BGC-823 cells, and bound endogenous DNMT3Ab and Snail were detected by Western blot. **b** The expression of *E-cadherin* was measured by qPCR in DNMT3Ab-tranfected MKN45 and BGC-823 cells transfected with Snail siRNA (*P < 0.05). **c** The methylation levels at the *E-cadherin* promoter were measured by Q-MSP in DNMT3Ab-tranfected MKN45 and BGC-823 cells transfected with Snail siRNA (*P < 0.05, **P < 0.01). **d** The binding of DNMT3Ab and H3K9me2, H3K27me3 status at the *E-cadherin* promoter were detected by ChIP-qPCR in DNMT3Ab-transfected MKN45 cells transfected with Snail siRNA (*P < 0.05). **e** Bar graphs depicting the migration and invasion rates of DNMT3Ab-tranfected MKN45 cells with Snail siRNA expression (**P < 0.01). The number of cells that migrated or invaded was counted in five fields. The migration and invasion rates are presented as the number of cells per field. **f** The migration rates of DNMT3Ab-tranfected MKN45 cells after Snail silencing were compared via wound healing assay. Microscopic observation was conducted at 0 and 36 h after scratching the surface of a confluent layer of cells

Snail is a transcriptional repressor that regulates the expression of *E-cadherin* during EMT [30]. To investigate the interaction between DNMT3Ab and Snail in *E-cadherin* regulation, immunoprecipitation (IP) assays showed that DNMT3Ab, but not DNMT3Aa, directly interacted with Snail (Fig. 6a and Supplementary Figure S6a). Meanwhile, we found that the binding of Snail to the *E-cadherin* promoter was increased in DNMT3Ab-transfected MKN45 and BGC-823 cells (Supplementary Figure S6b). Next, increased *E-cadherin* expression accompanied by de-methylation at the *E-cadherin* promoter was found in DNMT3Ab-transfected cells after silencing Snail expression (Fig. 6b, c). Meanwhile, ChIP assays revealed that the occupancy of DNMT3Ab, H3K9me2 and H3K27me3 at the *E-cadherin* promoter was significantly decreased upon silencing of Snail expression in DNMT3Ab-transfected cells (Fig. 6d). An interaction between the histone methyltransferases G9a or EZH2 and Snail was found, explaining the changes in H3K9me2 and H3K27me3 levels (Supplementary Figure S6c and d). To further evaluate whether the aggressive phenotype of GC cells induced by DNMT3Ab is dependent on Snail levels, we observed that silencing Snail reversed the migration and invasion of DNMT3Ab-transfected cells (Fig. 6e, f). These results suggested that Snail is at least partly required for the ability of DNMT3Ab to coordinate histone methylations in the modulation of *E-cadherin* expression and cell motility.

### DNMT3Ab alters the metastasis-associated gene expression profile

**Fig. 7:**
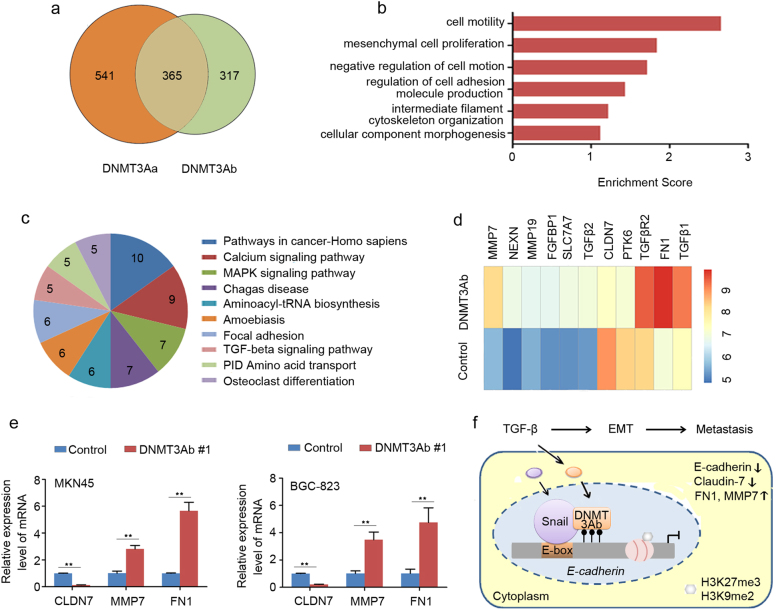
DNMT3Ab alters the expression of some metastasis-related molecular. **a** Venn diagram showing the genes affected by DNMT3Ab overexpression. **b** The bar plot shows the top six most significantly enriched biological process GO terms and the corresponding scores. Enrichment score equals (−log_10_ (*P*-value)). **c** Pathway analysis displaying the clustering of the 317 genes specific to DNMT3Ab into functional groups. **d** Heatmap clustering of microarray results showing that DNMT3Ab affects the expression of several metastasis-related genes. **e** qPCR was used to confirm CLDN7, MMP7 and FN1 mRNA expression in DNMT3Ab-overexpressing MKN45 and BGC-823 cells (**P < 0.01). **f** A model illustrating that DNMT3Ab-mediated DNA methylation and associated histone methylation causes the repression of *E-cadherin* and subsequent induction of EMT in a Snail-dependent manner

To explore whether DNMT3Ab regulation is specific to GC cell motility or the process of EMT in general, gene expression profiles analysis was performed on DNMT3Ab-transfected and control MKN45 cells. We compared the DNMT3Ab microarray data with our previous DNMT3Aa microarray data (No. GSE71020) and identified 317 genes specific to DNMT3Ab (Fig. 7a). Of these genes, gene ontology (GO) and pathway analysis showed that cell motility was the most highly impacted biological process (Fig. 7b) and that TGF-β was one of the top ten impacted cellular signalling pathways (Fig. 7c). Notably, the dysregulated genes in these pathways included *MMP7*, *NEXN*, *MMP19*, *FGFBP1*, *claudin-7* (*CLDN7*), *PTK6* and *fibronectin1* (*FN1*), which have been implicated in tumour cell invasion or the EMT process (Fig. 7d). Furthermore, we validated this microarray data and found that higher DNMT3Ab expression significantly altered the levels of *CLDN7*, *MMP7* and *FN1* mRNA (Fig. 7e). Collectively, these results indicate that DNMT3Ab is involved in controlling GC cell migratory and invasive abilities by regulating the expression of multiple metastasis-related genes.

## Discussion

Aberrant DNA methylation is recognised as one of the most important events in cancer metastasis. Currently, our understanding of the role of DNMTs in solid tumours, especially in GC, remains largely unknown. Here, we identified a novel function of DNMT3Ab in GC metastasis. DNMT3Ab is overexpressed in GC tissues, and its overexpression promotes tumour cell migration, invasion and metastasis by inducing EMT and increasing cell motility. This biological effect is mediated through the epigenetic silencing of *E-cadherin*, which is facilitated by the recruitment of DNMT3Ab to its promoter. Meanwhile, DNMT3Ab together with H3K9me2 and H3K27me3 contributed to *E-cadherin* regulation in a Snail-dependent manner. In addition, microarray data showed that several EMT-related genes are targets of DNMT3Ab in GC cells. A schematic model briefly summarising our work is shown in Fig. 7f.

This study provides three insights into the epigenetic programme in metastatic GC. First, we show that DNMT3Ab serves as an important regulator of GC cell metastasis. DNMT3A has been implicated in the dysregulation of several cellular processes in cancers [31–34]. Silencing DNMT3A results in dramatic inhibition of melanoma growth and metastasis [31]. Similarly, DNMT3A-mediated methylation reduces *PTEN* expression and promotes hepatocellular carcinoma cell proliferation and colony formation [32]. Although the function of DNMT3A as a whole has been described in tumour progression, its isoforms exhibit different sub-nuclear localisation and expression patterns, suggesting that each DNMT3A isoform may have unique functional specificity [23, 35]. Recently, a novel mouse model showed that DNMT3ab function is essential for genomic imprinting and is involved in genome integrity and cognitive diseases [36–38]; thus, we investigated the function of DNMT3Ab in the context of human tumours. Here, we found that DNMT3Ab was overexpressed in GC tumour tissues and indicated an unfavourable prognosis in patients, whereas DNMT3Aa expression showed no correlation with TNM stage, lymph node metastasis and overall survival time. Local invasion is known as one of the early steps in tumour metastasis. In vitro and in vivo studies found that DNMT3Ab promoted GC cell motility and increased tumour-nodule formation in mice. Interestingly, DNMT3Ab, but not DNTM3Aa, induced EMT, which is a primary characteristic of tumour-initiating cells (T-ICs) [39]. GC cells with elevated DNMT3Ab expression may contain more potential T-ICs and thus carry greater metastatic potential. Further studies into the stage- or tissue-specific roles of DNMT3A isoforms are necessary to determine whether different isoforms affect specific malignancies.

Second, we identify a critical molecular mechanism underlying the epigenetic regulation of EMT in GC. EMT is a key process in multicellular organisms [40]. Aberrant activation of EMT endows gastric epithelial cells with fewer epithelial and more mesenchymal features, which is triggered by the establishment of a unique gene expression pattern through epigenetic regulations [41]. *E-cadherin* is considered a typical epithelial marker during EMT, and the loss of *E-cadherin* has been found in gastrointestinal tumours [42]. Our previous study mainly focused on the cross-talk between DNMT3A and its upstream regulators and indicated that dysregulation of the DNMT3A-miR-29b/c axis led to the epigenetic silencing of *E-cadherin*. However, DNMT3A was evaluated only as a whole because its isoforms share the same 3′-UTR bound by miR-29b/c. Here, we extended our previous work and found that DNMT3Ab may be a major isoform involved in the induction of EMT by directly binding to the *E-cadherin* promoter and enhancing DNA methylation. Silencing DNMT3Ab partially blocked the TGF-beta-mediated EMT process. However, TGF-beta most likely induced DNMT3Ab expression in an indirect manner because DNMT3Ab has not been detected as a direct transcriptional target of the TGF-beta signalling pathway in GC. Potential mediators may play an important role in the TGF-beta-DNMT3Ab axis. In addition, a recent study found that DNMT3Ab and HDAC2 could reside together in a complex [37], indicating that DNMT3Ab could couple with histone modifications for the transcriptional regulation of genes [7]. Indeed, we showed that the levels of H3K9me2 and H3K27me3 at the *E-cadherin* promoter decreased with the reduction in DNMT3Ab, which indicated that DNMT3Ab-mediated DNA methylation is intimately linked to histone methylations. Intriguingly, DNMT3Ab lacks a DNA-binding sequence at the N-terminus of its protein, suggesting that a mediator is required to assist DNMT3Ab binding to target DNA. Snail is one key trigger of EMT and mediates its effects by binding directly to the E-box of *E-cadherin* promoter [43]. Thus, we further found that Snail is essential for the recognition of the *E-cadherin* promoter by DNMT3Ab with H3K9me2 and H3K27me3. Silencing Snail dramatically attenuated the DNMT3Ab-enhanced migration and invasion of GC cells, suggesting that Snail provides a platform for the binding of DNMT3Ab and histone methylation at the *E-cadherin*.

Third, we provide evidence for DNMT3Ab as a potential therapeutic target. Drugs modifying DNA methylation status have been used alone or in combination to achieve therapeutic outcomes [44]. Of these drugs, 5-Aza is a powerful inhibitor of DNA methylation. However, its clinical efficacy remains unsatisfactory, and it is associated with problems including, but not limited to, whether global genomic methylation may cause genomic instability [45]. Therefore, a superior approach is to selectively inhibit different DNMT family members with specific inhibitors. Here, we found that DNMT3Ab expression was positively correlated with TNM stage and lymph node metastasis and revealed that DNMT3Ab is an independent risk factor for reduced patient survival after curative resection. In addition, our microarray data indicated that DNMT3Ab regulated a wide array of tumour EMT-related genes in GC. Multiple oncogenic signalling pathways mediated by TGF-β or MAPK, for example, respond to DNMT3Ab upregulation, suggesting that DNMT3Ab may be a useful target to prevent local invasion and distant metastasis. Thus, detection of DNMT3Ab levels could facilitate the selection of a specific inhibitor to treat metastatic GC with high DNMT3Ab expression.

In summary, our present study highlights the importance of DNMT3Ab in the regulation of GC metastasis and its prognostic significance. We provide a potential direction in exploring the function of DNMT3A isoforms. These findings add diverse roles and mechanistic insight into our understanding of DNMT3A, define DNMT3Ab as a potential target for predicting clinical outcomes for patients, and may potentially lead to the development of novel strategies aimed at the prevention or early treatment of GC progression.

## Materials and methods

### Clinical samples

One-hundred and thirty GC tissues were obtained from the Affiliated Nanjing First Hospital between 2005 and 2012. Sixty-six pairs of GC tissue and adjacent non-tumour tissue were collected from patients at the Third Affiliated Hospital of Harbin Medical University between 2011 and 2013. All the patients enroled in this study were newly diagnosed and had not received any previous treatment. This study was reviewed and approved by the Committee for Ethical Review of Research at the Affiliated Nanjing First Hospital and the Third Affiliated Hospital of Harbin Medical University in China, and the patients provided written informed consent forms. The methods were carried out in accordance with approved guidelines.

### Cell lines and cell culture

The GES-1 immortalised normal human gastric cell line was preserved in our laboratory. The AGS, BGC-823, MCG-803, MKN45, MKN28, NCL-N87 and SGC-7901 human GC cell lines were obtained from the Cell Bank of the Chinese Academy of Science in 2012. Immediately after receipt, the cell lines were expanded and frozen such that they could be revived every 3 to 4 months. The cell lines were tested and found negative for mycoplasma contamination. All the cell lines were used at passages 3 to 10 and maintained in RPMI-1640 medium supplemented with 10% foetal bovine serum (Invitrogen, Carlsbad, CA), 100 U/ml penicillin and 100 mg/ml streptomycin (Invitrogen) in a humidified incubator at 37 °C and 5% CO_2_.

### Real-time quantitative PCR (qPCR)

Total RNA was extracted from the cells and tissues using TRIzol reagent (Invitrogen, Carlsbad, CA) according to the manufacturer’s instructions. qPCR was carried out by SYBR Premix Ex Taq (Takara, Dalian, China) using the StepOne real-time PCR System (Applied Biosystems). Relative expression was evaluated using the comparative CT method and normalised to human β*-*actin mRNA expression. The experiments were independently repeated at least three times. The primer sequences for each gene are shown in Supplementary Table S3.

### Gene expression profiles and data analysis

An Affymetrix GeneChip Human Transcriptome Array 2.0 was used for microarray analysis. Microarray hybridisation was performed by Biotechnology Corporation (Shanghai, China) using standard Affymetrix procedures. Raw microarray data were acquired using GCOS1.2 software from Affymetrix and preprocessed using robust multiarray analysis (RMA) with log base 2 (log2) transformations. GO analysis was used to identify the GO terms associated with the differentially expressed mRNAs.

### Immunofluorescence staining

For immunofluorescence staining, GC cells were incubated with the primary antibody (mouse anti-E-cadherin, 1:100, Abcam or rabbit anti-Vimentin, 1:100, Santa Cruz Biotechnology) overnight at 4 °C. After thorough washing, the cells were incubated with a mixture of Alexa Fluor 555-conjugated goat anti-mouse IgG and Alexa Fluor 647-conjugated goat anti-rabbit IgG (1:300, Invitrogen). For F-actin staining, cells were stained with Alexa Fluor 488-conjugated phalloidin (Invitrogen). Finally, DAPI (Invitrogen) was used to counterstain the cell nuclei. The fluorescent sections were observed, and images were captured with an LSM 700 confocal microscope.

### Chromatin immunoprecipitation

The ChIP assays were performed using an EZ-Magna ChIP G kit (Upstate Biotechnology, Lake Placid, NY, USA) following the manufacturer’s instructions. The final DNA extracts were qPCR amplified. The primers used to amplify the precipitated DNA fragments are listed in Supplementary Table S3.

### Immunoprecipitation

For IP assays, the cells were washed with cold PBS and lysed with cold lysis buffer at 4 °C for 30 min. Whole cell lysates were incubated with 1 µg of antibody together with 10 µl of Protein G Agarose (Invitrogen). After being washed with IP buffer, the IP’ed proteins were analysed by Western blot using the indicated antibodies.

### Animal experiments

Approximately 6 × 10^5^ cells were injected into female nude mice (4 weeks old) via the tail vein (*n* = 6 for each group). All the mice were euthanised after 6 weeks. The number of tumour nodules formed on the lung surfaces was counted. The lungs were excised and embedded in paraffin. All the procedures were conducted in accordance with the institutional standard guidelines of Southeast University for animal experiments.

### Statistical analysis

The levels of DNMT3Ab protein in tumour tissue from patients with GC and the paired adjacent non-tumour tissues were compared using Wilcoxon’s test. The correlations between protein expression and clinicopathological features were analysed using Pearson’s *χ*^2^ test for categorical variables. The Kaplan–Meier method and log-rank tests were used for survival analysis. Correlations between the expression levels of two molecules were analysed by Spearman’s test. The Cox proportional hazards model was used to determine the independent factors that influence survival based on the variables that were selected from the univariate analysis. Two-tailed Student’s *t*-test was used to compare the results for any two preselected groups accounting for variance, which were expressed as the mean ± S.D. of three independent experiments. *P*-values less than 0.05 were considered statistically significant. All the analyses were performed using SPSS software (version 16.0).

## Electronic supplementary material

Supplementary materials and methods

Supplementary Table

Supplementary figure legends

Figure S1

Figure S2

Figure S3

Figure S4

Figure S5

Figure S6
