# Supplementary material for: DNA methyltransferase 3A isoform b contributes to repressing E-cadherin through cooperation of DNA methylation and H3K27/H3K9 methylation in EMT-related metastasis of gastric cancer
Source: Oncogene. 2018 May 2;37(32):4358–71. doi: 10.1038/s41388-018-0285-1 (PMC6085280; doi:10.1038/s41388-018-0285-1)
Supplement: Supplementary file 1 — Supplementary materials and methods [file 41388_2018_285_MOESM1_ESM.docx]

**Transfection**

A pIRESpuro/Myc-DNMT3Ab construct was kindly provided by Dr. Gang-Ning Liang of the Jane Anne Nhol Division of Hematology, at Norris Cancer Center (Los Angeles, CA, USA). Expression plasmids were transfected into MKN45 and BGC-823 cells using Lipofectamine 2000 (Invitrogen, Carlsbad, CA, USA) according to the manufacturer’s instructions. Cells were selected with medium containing 0.4µg/ml puromycin (Clontech, USA) for 30 days, and monoclonal cell lines were established. Vector-based short hairpin RNAs (shRNAs) against DNMT3Ab N-terminal were transfected into MKN28 cells using Lipofectamine 2000 according to the manufacturer’s instructions and selected with medium containing 400μg/μl G418 (Gibco, Gaithersburg, MD, USA) for 30 days. A shRNA with a sequence that did not match any known human gene was used as the negative control. Oligonucleotide sequence of shRNAs was designed by GenePharma Company (Shanghai, China) and shown in Supplementary Table S4.

**RNA interference**

siRNA was transfected into cells in 6-well plates using Lipofectamine 2000 according to the manufacturer’s instructions (Invitrogen, Carlsbad, CA). siRNA (20nM) against Snail or DNMT3Aa were synthesized by the GenePharma Company (Shanghai, China). Gene silencing was measured by qPCR analysis 48 hours after transfection. Oligonucleotide sequence of siRNAs was shown in Supplementary Table S4.

**Antibodies and Western blot**

DNMT3Ab antibody was purchased from Millipore (Cat.07-2050). Rabbit anti-DNMT1 (ab109981), anti-DNMT3Aa (ab2850), anti-DNMT3B (ab16049), anti-E-cadherin (ab40772), anti-G9a (ab40542) and mouse anti-*c*-Myc (ab32) were purchased from Abcam. Rabbit anti-*β*-catenin (#8480), anti-N-cadherin (#4061), anti-PCNA (#13110), anti-EZH2 (#4905), anti-H3K9me2 (#4658) and anti-H3K27me3 (#9733) were purchased from Cell Signaling Technology. Mouse anti-Vimentin (sc-6260) was purchased from Santa Cruz Biotechnology. Goat anti-Snail (AF3639) was purchased from R&D system. Mouse anti-*β*-actin was obtained from Sigma-Aldrich. Proteins were detected with Super Signal Chemiluminescence Substrate (Pierce, USA).

**Immunohistochemistry (IHC)**

Paraffin-embedded tissue blocks were sectioned for IHC. In brief, the deparaffinized and hydrated sections were placed in a pressure cooker with 0.01mol/L citric acid buffer solution and boiled for 5 minutes to retrieve antigenic epitopes. The slides were incubated with a 1:100 dilution of antibody in a moist chamber at 4°C overnight. The slides were incubated with biotinylated secondary antibody (1:200, Vector Laboratories) and then with peroxidase-conjugated streptavidin for 30 minutes at 37°C. Finally, the sections were reacted with DAB or alkaline phosphatase. The sections were photographed with a Leica DM2500 photomicroscope equipped with a video camera controlled by Leica QWin Lite software. Protein expression was evaluated following the method of previous study.^1^

**Bisulfite sequencing (BGS) and promoter methylation analysis**

Genomic DNA was extracted from cells using the phenol-chloroform method. Bisulfite treatment was performed using a CpGenomeTM Universal DNA Modification Kit (Millipore, USA), following the manufacturer’s instructions. For BGS analysis, modified DNA was amplified, and PCR products were gel-purified and sub-cloned into a pMD19-T vector system (TAKARA, Japan). Ten colonies were sequenced to assess the degree of methylation at each CpG site. Moreover, modified DNA was amplified to determine the methylation status of the promoter region of target gene by quantitative real-time methylation-specific PCR (Q-MSP) as described previously. ^2^ The experiments were independently repeated at least three times. The primers used are listed in Supplementary Table S3.

**Cell foci formation**

A focus formation assay was performed by seeding 1×10^3^ cells in a 6-well plate. The surviving colonies (>50 cells per colony) were counted following crystal violet (Invitrogen, Carlsbad, CA, USA) staining. All experiments were independently repeated at least three times.

**Wound healing, migration and invasion assays**

A scratch wound was generated in confluent cell monolayers in six-well plates using a 200μl pipette tip. The cells were then washed with fresh medium to remove floating cells, and wound closure was observed after 24 or 36 hours and photographed under a microscope. The migratory and invasive potential of the transfected cells were evaluated with a transwell assay. In the migration assay, the cells were cultured in 200μl medium with 1% fetal bovine serum in the upper chamber of a non-coated transwell insert. In the lower chamber, 600μl medium with 10% fetal bovine serum was used as a chemoattractant to encourage cell migration. In the invasion assay, the upper chamber of the transwell inserts was coated with 50μl of 1.0 mg/ml Matrigel, and the cells were plated in the upper chamber of the Matrigel-coated transwell insert (Millipore, Billerica, MA, USA). After 24-hour incubation, the non-migrating or non-invading cells were gently removed with a cotton swab. All of the cells were stained with 0.1% crystal violet, and the cells in five fields were counted using an inverted microscope. The experiments were independently repeated three times.

**Accession codes**

DNMT3Aa (NM_175629), DNMT3Ab (NM_153759)

**REFERENCES**

1. Zhang A, Yu H, He Y, Shen Y, Pan N, Liu J*, et al.* The spatio-temporal expression of MHC class I molecules during human hippocampal formation development. *Brain Res* 2013; **1529**: 26-38.

2. Chan MW, Chu ES, To KF, Leung WK. Quantitative detection of methylated SOCS-1 , a tumor suppressor gene, by a modified protocol of quantitative real time methylation-specific PCR using SYBR green and its use in early gastric cancer detection. *Biotechnol Lett* 2004; **26**: 1289-1293.
