# Supplementary material for: DNA methyltransferase 3A isoform b contributes to repressing E-cadherin through cooperation of DNA methylation and H3K27/H3K9 methylation in EMT-related metastasis of gastric cancer
Source: Oncogene. 2018 May 2;37(32):4358–71. doi: 10.1038/s41388-018-0285-1 (PMC6085280; doi:10.1038/s41388-018-0285-1)
Supplement: Supplementary file 2 — Supplementary Table [file 41388_2018_285_MOESM2_ESM.docx]

Table S1 Clinicopathological correlation of DNMT3Aa expression in GC cases

| Feature | T > N | T ≤ N | *P*-value |
| --- | --- | --- | --- |
| Age | 58.39±10.47 | 59.79±12.30 | 0.616 |
| Gender (n=66) |  |  |  |
| Female | 10 | 7 |  |
| Male | 27 | 22 | 0.790 |
| Diameter(n=64) |  |  |  |
| ≤ 5cm | 24 | 12 |  |
| > 5cm | 12 | 16 | 0.057 |
| Lauren (n=56) |  |  |  |
| Diffuse type | 18 | 12 |  |
| Intestinal type | 15 | 11 | 0.861 |
| Histologic grade (n=63) |  |  |  |
| Poor | 26 | 10 |  |
| Moderate | 10 | 17 |  |
| High ^a^ | 0 | 0 | **0.005*** |
| TNM staging (n=65) |  |  |  |
| Stage I / II | 14 | 11 |  |
| Stage III / IV | 22 | 18 | 0.937 |
| Vascular invasion (n=60) |  |  |  |
| Yes | 9 | 1 |  |
| No | 24 | 26 | **0.015*** |
| Lymph node metastasis  (n=66) |  |  |  |
| Yes | 29 | 23 |  |
| No | 8 | 6 | 0.927 |

* Significant differences are shown in bold. N: Non-tumour tissues; T: tumour tissues.

**^a^** No cases in this pathological classification

Table S2 Clinicopathological correlation of DNMT3Ab expression in IHC cases

| Feature  (n=130) | Low expression  (+) | High expression  (++，+++) | *P*-value |
| --- | --- | --- | --- |
| Age | 64.02±10.39 | 64.75±12.20 |  |
| Gender |  |  |  |
| female | 21 | 15 |  |
| male | 43 | 51 | 0.199 |
| Diameter |  |  |  |
| ≤ 5cm | 30 | 39 |  |
| > 5cm | 34 | 27 | 0.163 |
| Lauren |  |  |  |
| Diffuse type | 53 | 55 |  |
| Intestinal type | 11 | 11 | 0.937 |
| Histologic grade |  |  |  |
| Poor | 34 | 24 |  |
| Moderate | 26 | 39 |  |
| High | 4 | 3 | 0.109 |
| TNM staging |  |  |  |
| Stage I / II | 4 | 16 |  |
| Stage III / IV | 60 | 50 | **0.004*** |
| Lymph node metastasis |  |  |  |
| Yes | 11 | 22 |  |
| No | 53 | 44 | **0.034*** |
| *Significant differences are shown in bold. | | | |

Table S3 Primers used in this study

| Gene | Sequences | |
| --- | --- | --- |
|  | sense | antisense |
| For qPCR |  |  |
| E-cadheirn | TTCTGTGAGAGGAATCCA | GTGTTAGTTCTGCTGTGA |
| DNMT3Ab | GCGTGGATCGTAGCCTGAAA | TCCTCCACCTTCTGAGACTC |
| DNMT3Aa | AACAGAAGGAGACCAACATCG | CGCTTGCTGATGTAGTAGGG |
| CLDN7 | GTAGCTTGCTCCTGGTAT | AACTCATACTTAATGTTGGTAGG |
| MMP7 | CAGTGATGTATCCAACCTAT | CAATCCAATGAATGAATGAATG |
| FN1 | GAATATGTAGTGAGTGTCT | AGAGTTGGCAGTAATATC |
| For Q-MSP |  |  |
| E-cadheirn | TAGTTCGGTTCGATTCGATC | GAATACGTCCCTCGCAAAT |
| β-actin | TGGTGATGGAGGAGGTTTAGTAAGT | AACCAATAAAACCTACTCCTCCCTTAA |
| For BGS |  |  |
| E-cadheirn | TTTAGTAATTTTAGGTTAGAGGGTTAT | AATACCTACAACAACAACAACAAC |
| For ChIP |  |  |
| E-cadheirn | CCCTCAGCCAATCAGCGGTA | GAGCGGGCTGGAGTCTGAAC |

Table S4 siRNA or shRNA sequences used in this study

| Gene | Sequences(5’-3’) |
| --- | --- |
| DNMT3Ab shRNA #1 | GATCCCCCGTGGATCGTAGCCTGAAATTCAAGAGA  TTTCAGGCTACGATCCACGTTTTTGGAAA |
| DNMT3Ab shRNA #2 | GATCCCCCTGAAAGACGAGTGTGATATTCAAGAGA TATCACACTCGTCTTTCAGTTTTTGGAAA |
| DNMT3Ab siRNA #1 | CGTGGATCGTAGCCTGAAA |
| DNMT3Ab siRNA #2 | CTGAAAGACGAGTGTGATA |
| DNMT3Aa siRNA #1 | CCAACATCGAATCCATGAA |
| DNMT3Aa siRNA #2 | GCTCAACACCGGGATCTAT |
| Snail siRNA #1 | CCCACTCAGATGTCAAGAA |
| Snail siRNA #2 | CCACCCTCACCGGCTCCTT |
