# Supplementary material for: DNA methyltransferase 3A isoform b contributes to repressing E-cadherin through cooperation of DNA methylation and H3K27/H3K9 methylation in EMT-related metastasis of gastric cancer
Source: Oncogene. 2018 May 2;37(32):4358–71. doi: 10.1038/s41388-018-0285-1 (PMC6085280; doi:10.1038/s41388-018-0285-1)
Supplement: Supplementary file 3 — Supplementary figure legends [file 41388_2018_285_MOESM3_ESM.docx]

**Supplementary Figure legends**

**Figure S1.** (**a, b**) The relative protein levels of DNMT3Aa or DNMT3Ab in 66 paired adjacent non-tumour (N) tissues and GC (T) tissues were divided into three groups (T>N, T=N and T<N) based on relative expression scores of greater than or less than 2-fold. The number of cases is shown for every group. (**c**) The expression of DNMT3Aa was detected by IHC staining in 130 GC tissues. (+) denotes low expression, (++, +++) denotes high expression. (**d**) Kaplan-Meier analysis of the correlation between DNMT3Aa expression and overall survival in 130 patients with GC (**P*<0.05).

**Figure S2.** (**a**) Screening of protein levels in immortalized normal GES-1 and seven GC cell lines by western blot. *β*-actin was used as a loading control (top). The band intensities were quantified and normalized to *β*-actin intensities with *ImageJ* software (bottom). (**b**) Bar graphs depict the migration and invasion of GC cell lines. The number of cells that migrated or invaded was counted in five fields. The migration and invasion rates are presented as the number of cells per field. (**c**) Generation of DNMT3Ab-tranfected stable cell lines. The efficiency of DNMT3Ab overexpression in MKN45 and BGC-823 cells was detected by western blot analysis. *β*-actin was used as a loading control (top). The band intensities were quantified and normalized to *β*-actin intensities with *ImageJ* software (bottom). (**d**) Generation of DNMT3Ab-knockdown stable cell lines. The efficiency of DNMT3Ab deficiency in MKN28 cells was detected by western blot analysis. The protein expression of DNMT3Aa was shown after the knock-down of DNMT3Ab. *β*-actin was used as a loading control (top). The band intensities were quantified and normalized to *β*-actin intensities with *ImageJ* software (bottom).

**Figure S3.** (**a**) The cell migration rates of DNMT3Ab-tranfected MKN45 and BGC-823 cells were shown by wound healing assays. Microscopic observation was completed 0, 24, and 36 hours after scratching the surface of a confluent layer of cells. (**b**) Representative images of foci formation in DNMT3Ab- tranfected MKN45 cells (top). Colonies were counted, and the results are depicted in a bar chart (bottom). (**c**) The Cell migration rates of DNMT3Ab knockdown MKN28 cells were shown by wound healing assays. Microscopic observation was completed 0, 24, and 36 hours after scratching the surface of a confluent layer of cells. (**d**) Representative images (left) and relative bar graphs (right) depicting the migration and invasion of DNMT3Ab-knockdown MCG-803 cells. The number of cells that migrated or invaded was counted in five fields. The migration and invasion rates are presented as the number of cells per field (***P*<0.01). (**e**) The protein expression of DNMT3Aa and DNMT3Ab was shown after the knock-down of DNMT3Ab by siRNAs. *β*-actin was used as a loading control. (**f**) Relative bar graphs depicting the migration and invasion of DNMT3Ab-knockdown MKN45 and BGC-823 cells. The number of cells that migrated or invaded was counted in five fields. The migration and invasion rates are presented as the number of cells per field. (**g**) The expression of epithelial markers (E-cadherin and *β*-catenin), as well as mesenchymal markers (Vimentin and N-cadherin) were detected by Western blot in DNMT3Ab-knockdown MKN28 cells relative to that in control cells. (**h**) DNMT3Ab or negative control siRNAs were transiently transfected into MKN45 and BGC-823 cells. Cell morphology with no changes associated with the EMT phenotype.

**Figure S4.** (**a**) The baseline levels of DNA methylation on *E-cadherin* promoter among GC cell lines. (**b, c**) Methylation levels at the *E-cadherin* promoter were detected by Q-MSP assays in DNMT3Ab-knockdown MCG-803, MKN45 and BGC-823 cells (**P*<0.05, ***P*<0.01). (**d**) The binding of DNMT3Aa to the *E-cadherin* promoter was detected by ChIP in DNMT3Ab-transfected cells. (**e**) The relative expression of *E-cadherin* was detected in DNMT3Ab-knockdown MCG-803 cells by qPCR*. β*-actin was used as an internal control (**P*<0.05). (**f**) The protein expression of DNMT3Aa and DNMT3Ab was shown after the knock-down of DNMT3Aa by siRNAs. *β*-actin was used as a loading control. (**g**) qPCR showing the relative expression of *E-cadherin* in 24 paired clinical GC specimens. The value (defined as “fold change”) indicates the ratio of the *E-cadherin* mRNA expression levels in the GC tissues versus paired adjacent non-tumour tissues. (**h**) The correlation between DNMT3Aa and *E-cadherin* expression in 24 clinical samples (R=-0.018, *P*>0.05).

**Figure S5.** (**a**) MKN45 and BGC-823 were treated with TGF-*β* (2 ng/ml) for 3 days. The expression of DNMT1, DNMT3Aa, DNMT3Ab and DNMT3B was detected in these cells by western blot. *β*-actin was used as a loading control. (**b**) The binding of DNMT3Aa and DNMT3Ab to the *E-cadherin* promoter in MKN45 and BGC-823 cells after TGF-*β* treatment (**P*<0.05, ***P*<0.01). (**c**) Baseline levels of H3K9me2 and H3K27me3 status of *E-cadheirn* promoter in MKN45, BGC-823 and MKN28 cells.

**Figure S6.** (**a**) Endogenous DNMT3Aa and Snail were immunoprecipitated from MKN45 and BGC-823 cells, and bound endogenous DNMT3Aa and Snail were detected by western blot. (**b**) The binding of Snail to *E-cadherin* promoter was shown in DNMT3Ab-trafected cells (**P*<0.05, ***P*<0.01). (**c**) Endogenous G9a and Snail were immunoprecipitated from MKN45 and BGC-823 cells, and bound endogenous G9a and Snail were detected by western blot. (**d**) Endogenous EZH2 and Snail were immunoprecipitated from MKN45 and BGC-823 cells, and bound endogenous EZH2 and Snail were detected by western blot.
