# Supplementary figures and images for: DNA methyltransferase 3A isoform b contributes to repressing E-cadherin through cooperation of DNA methylation and H3K27/H3K9 methylation in EMT-related metastasis of gastric cancer
Source: Oncogene. 2018 May 2;37(32):4358–71. doi: 10.1038/s41388-018-0285-1 (PMC6085280; doi:10.1038/s41388-018-0285-1)

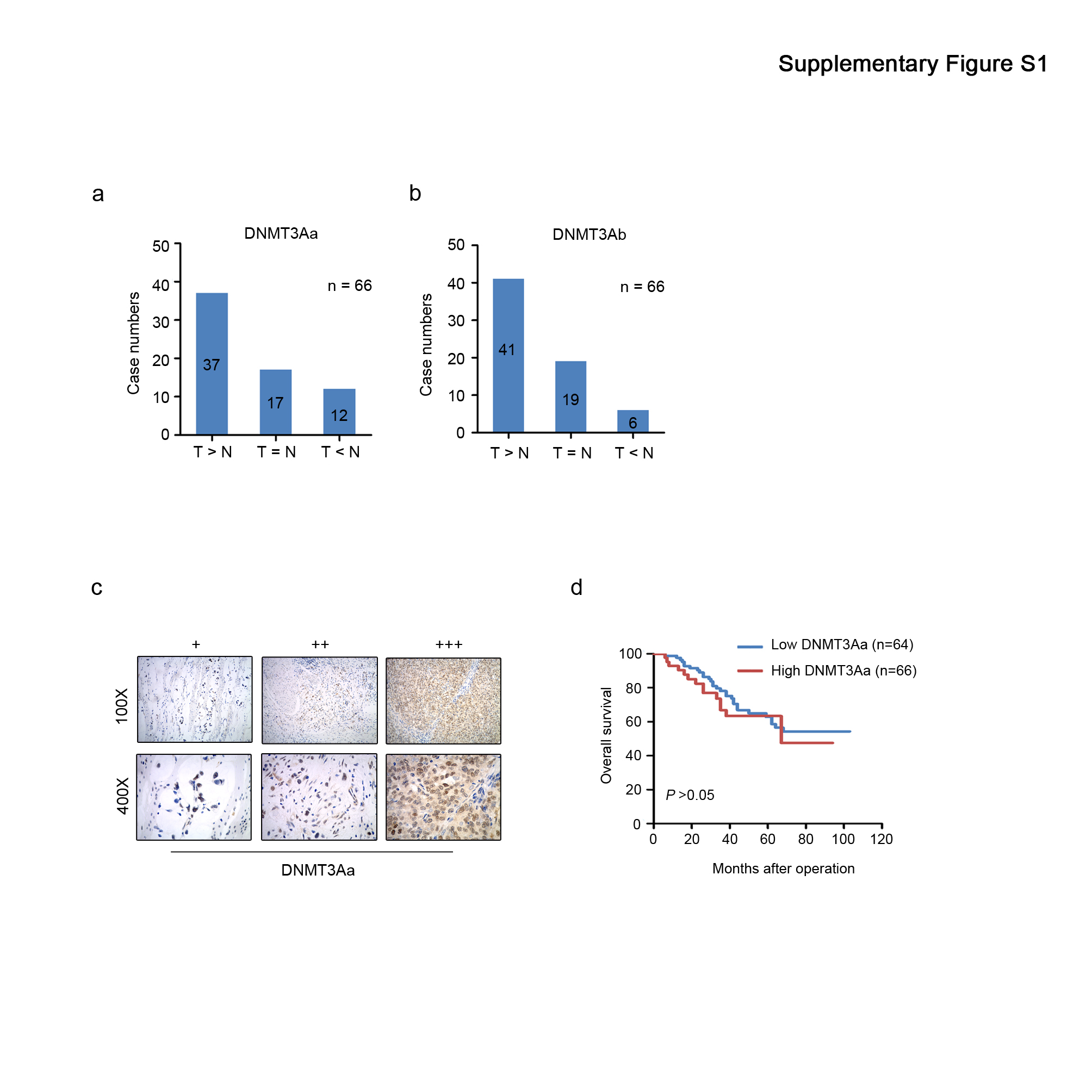

Supplement: Supplementary file 4 — Figure S1 [file 41388_2018_285_MOESM4_ESM.jpg]

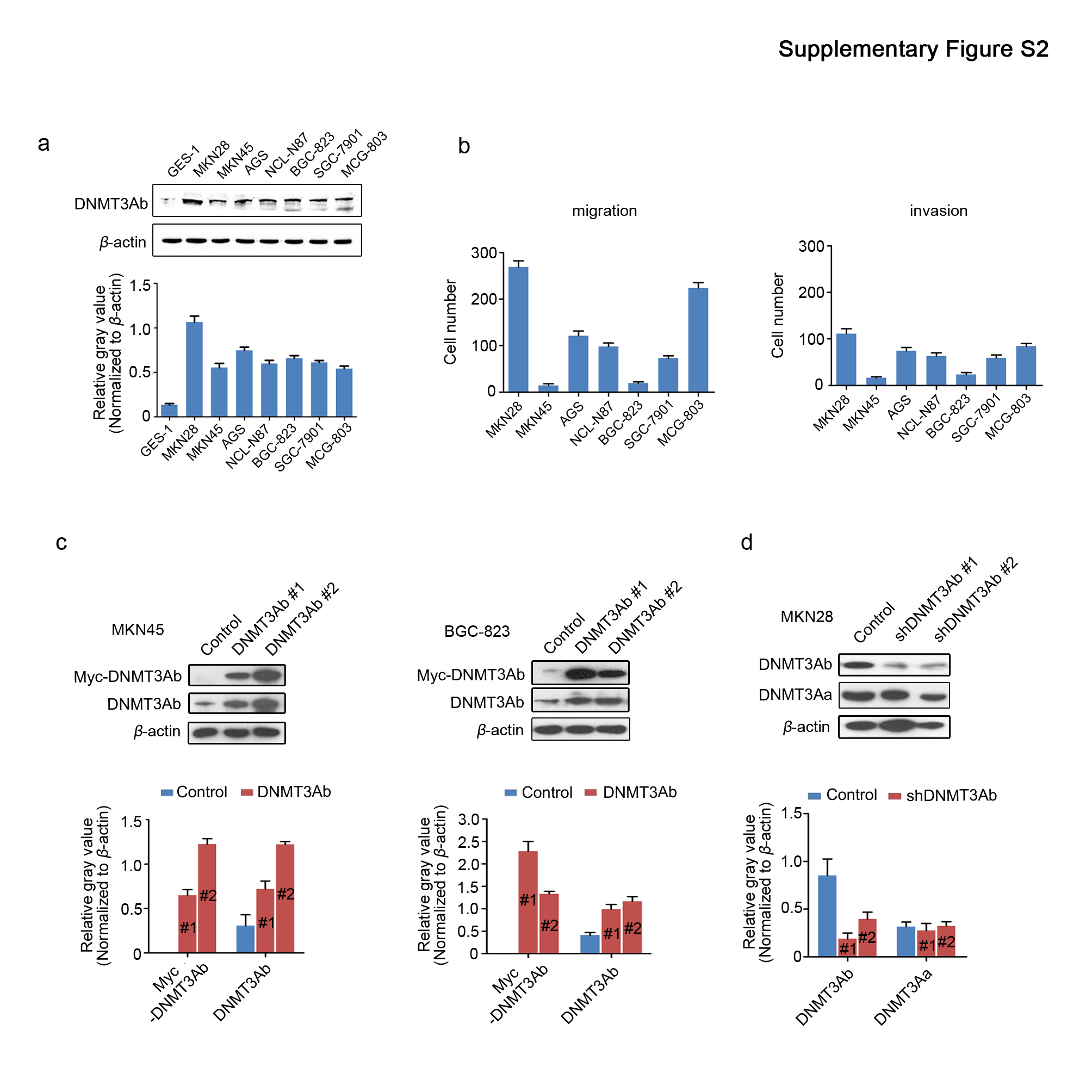

Supplement: Supplementary file 5 — Figure S2 [file 41388_2018_285_MOESM5_ESM.jpg]

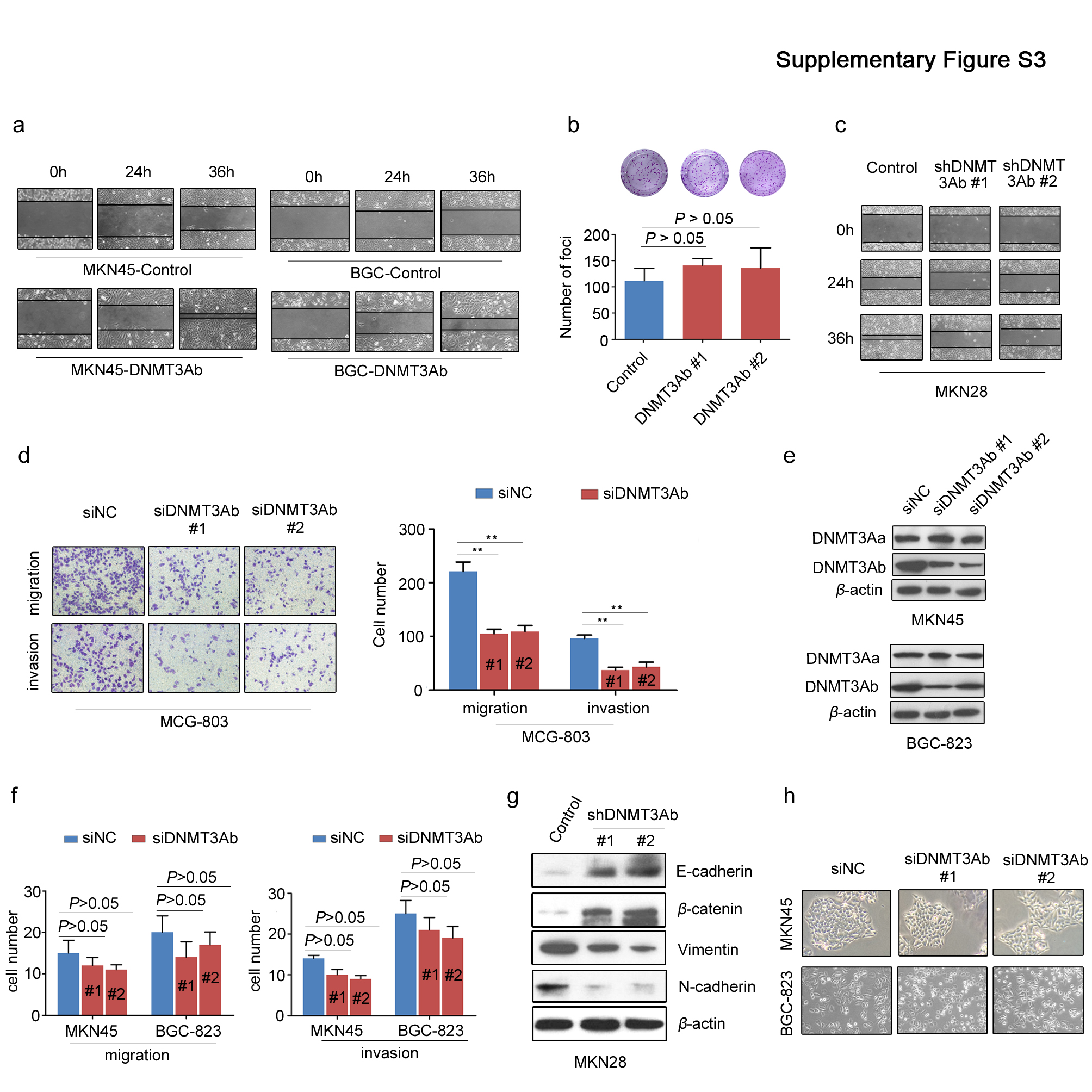

Supplement: Supplementary file 6 — Figure S3 [file 41388_2018_285_MOESM6_ESM.jpg]

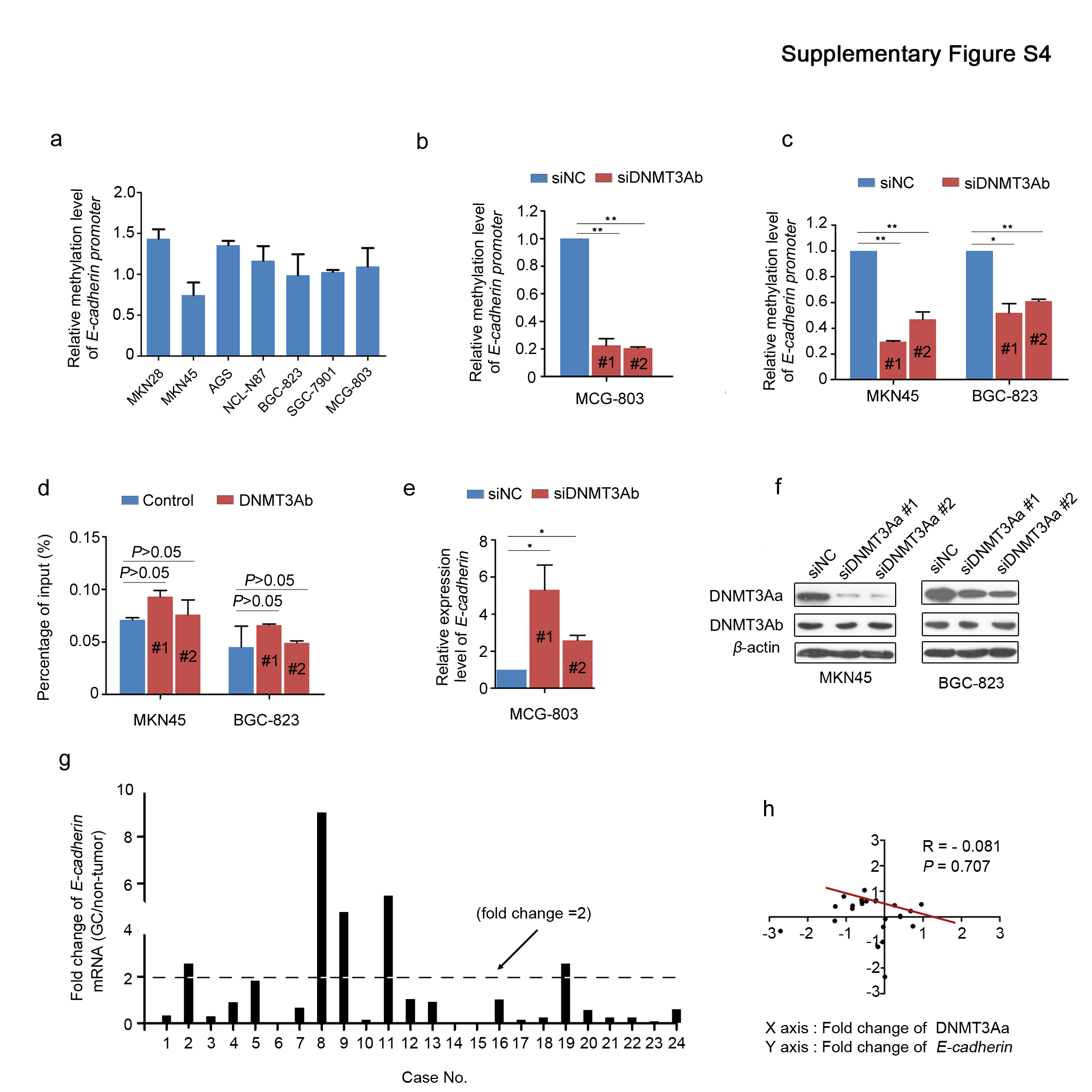

Supplement: Supplementary file 7 — Figure S4 [file 41388_2018_285_MOESM7_ESM.jpg]

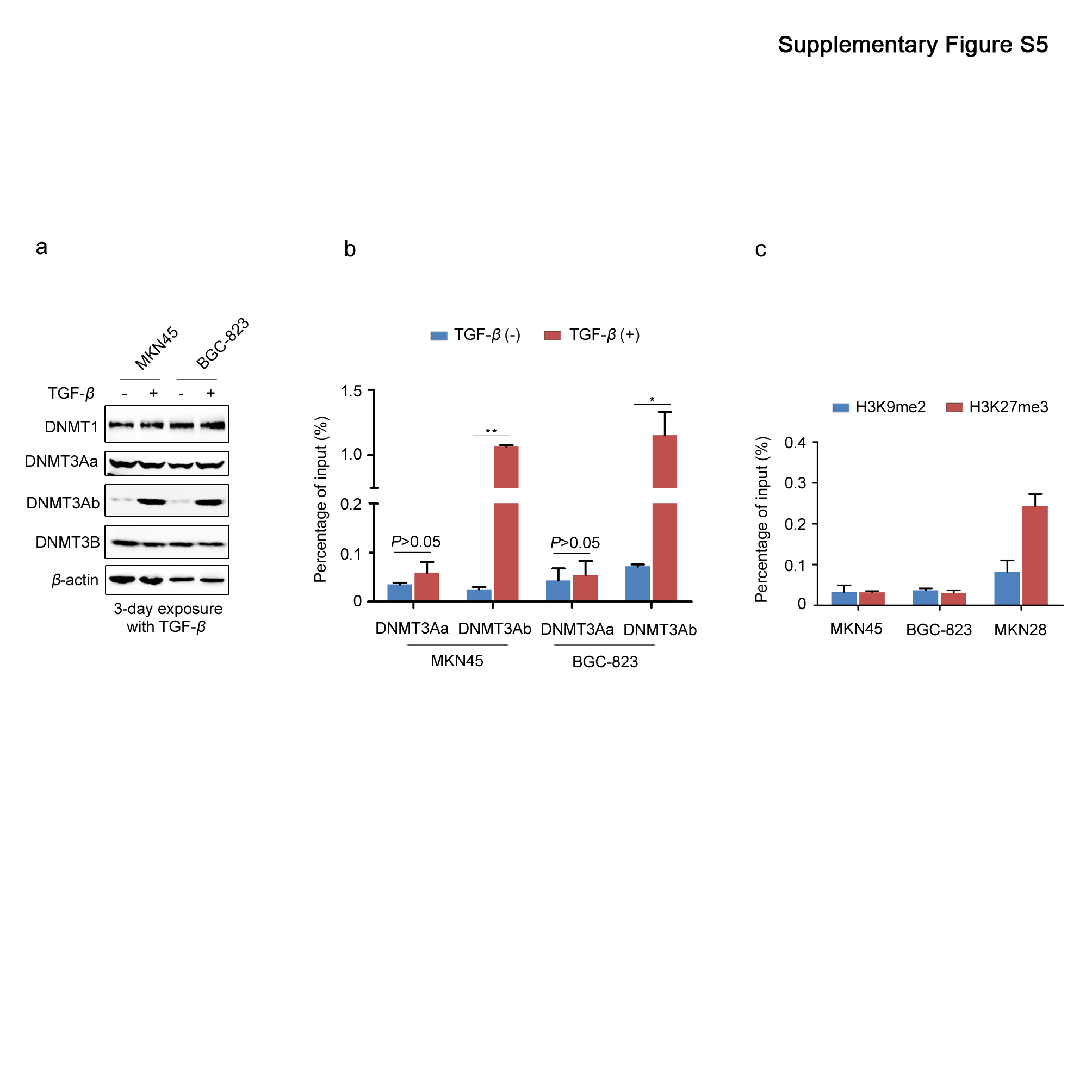

Supplement: Supplementary file 8 — Figure S5 [file 41388_2018_285_MOESM8_ESM.jpg]

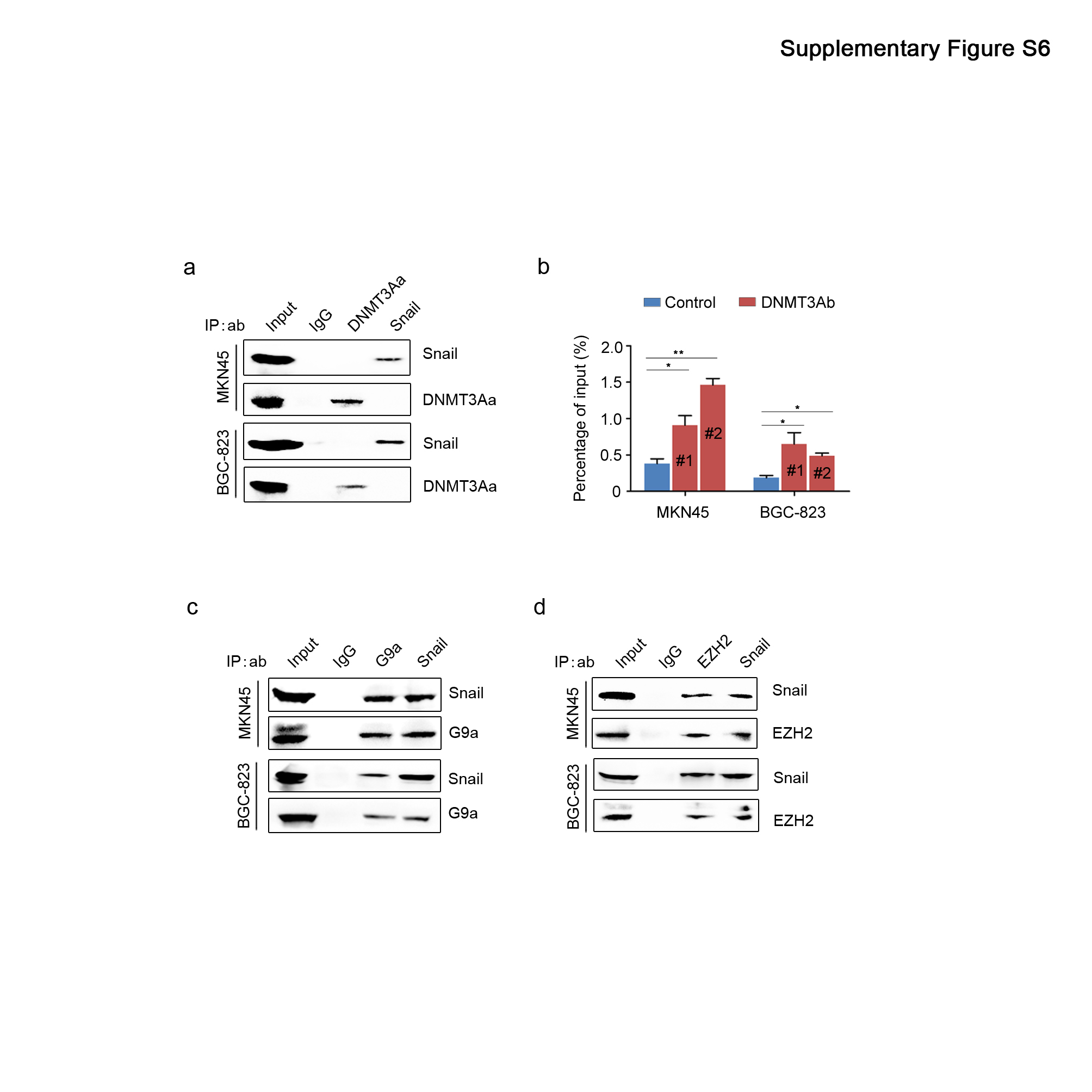

Supplement: Supplementary file 9 — Figure S6 [file 41388_2018_285_MOESM9_ESM.jpg]
